# Analysis for Clinical Effect of Virtual Windowing and Poking Reduction Treatment for Schatzker III Tibial Plateau Fracture Based on 3D CT Data

**DOI:** 10.1155/2015/231820

**Published:** 2015-02-12

**Authors:** Huafeng Zhang, Zhijun Li, Qian Xu, Yuan Zhang, Ke Xu, Xinlong Ma

**Affiliations:** ^1^Department of Orthopedics, Tianjin Medical University General Hospital, No. 154 Anshan Road, Tianjin 300052, China; ^2^Tianjin University of Traditional Chinese Medicine, No. 88 Yuquan Road, Nankai, Tianjin 300193, China; ^3^Tianjin Hospital, No. 406 South JieFang Road, Tianjin 300210, China

## Abstract

*Objective*. To explore the applications of preoperative planning and virtual surgery including surgical windowing and elevating reduction and to determine the clinical effects of this technology on the treatment of Schatzker type III tibial plateau fractures. *Methods*. 32 patients with Schatzker type III tibial plateau fractures were randomised upon their admission to the hospital using a sealed envelope method. Fourteen were treated with preoperative virtual design and assisted operation (virtual group) and 18 with direct open reduction and internal fixation (control group). *Results*. All patients achieved primary incision healing. Compared with control group, virtual groups showed significant advantages in operative time, incision length, and blood loss (*P* < 0.001). The virtual surgery was consistent with the actual surgery. *Conclusion*. The virtual group was better than control group in the treatment of tibial plateau fractures of Schatzker type III, due to shorter operative time, smaller incision length, and lower blood loss. The reconstructed 3D fracture model could be used to preoperatively determine the surgical windowing and elevating reduction method and simulate the operation for Schatzker type III tibial plateau fractures.

## 1. Introduction

Fractures of the tibial plateau involve a major weight-bearing joint, and their successful treatment requires the restoration of joint congruity in order to preserve normal knee function. Tibial plateau fractures are commonly classified using the Schatzker method. Schatzker method of tibial plateau fractures is currently the most widely used and was the first to make the distinction between medial and lateral plateau fractures. In this study, we investigated Schatzker type III fractures, which are pure compression fractures that result in central depression of the lateral tibial plateau into the tibial metaphysis and usually occur in patients with osteoporosis [1].

Successful treatment of tibial plateau fractures mainly depends on anatomical reduction of the tibial plateau to restore articular surface continuity [2]. Preoperative planning was crucial for the successful reduction of these fractures. The creation of clear and accurate three-dimensional (3D) digital fracture models using 3D computed tomography (CT) reconstruction and the development of virtual surgery technology have greatly aided preoperative planning [3]. Furthermore, the meticulous planning enabled by these technologies has allowed minimally invasive fracture surgery and improved surgical safety and efficiency [4].

Computer-aided design surgery may help us to find the surgical problems in advance. Through the actual operation of application demonstrate the effectiveness of preoperative virtual surgery. The purpose of the present study was to compare preoperative virtual design and assisted operation with direct ORIF clinically and to show the advantage preoperative virtual design and assisted operation.

## 2. Materials and Methods

### 2.1. General Information

Between August 2011 and April 2013, 36 patients with Schatzker type III tibial plateau fractures were randomised upon their admission to the hospital using a sealed envelope method in Department of Orthopedics, Tianjin Medical University General Hospital. The sealed envelope method: the doctors involved in the clinical trials followed in the instruction of treatment plan according to the randomly generated treatment inside the sealed envelope. The inclusion criteria were (1) having closed and fresh fractures; (2) having Schatzker type III tibia plateau fractures, pure central depression; (3) being with mild or moderate osteoporosis, suitable for internal fixation; (4) having no nerve and vascular injury. The exclusion criteria were (1) being with other parts of the limb fracture; (2) being with pathological fractures, such as osteoclastoma and bone cyst; (3) being unable to cooperate with treatment because of mental disorder. Of the 36 patients, 4 refused to do the operation. The remaining 32 patients were available for analysis: 14 patients treated by preoperative virtual design and assisted operation (virtual group) and 18 patients with direct ORIF (control group). All participants provided written informed consent approved by the Hospital Human Research Ethics Committee.

Virtual groups: 4 cases were male and 10 cases were female. The average age was 46.1 years (range, 41–53 years). Accident injuries were 4 cases and falling down injuries were 10 cases. The average time from injury to the operation was 4.5 days (range, 2–10 days). Control group: 6 cases were male and 12 cases were female. The average age was 46.8 years (range, 40–56 years). Accident injuries were 5 cases and falling down injuries were 13 cases. The average time from injury to the operation was 4.3 days (range, 2–9 days). The independent Student's *t*-test was used to analyse the differences with age, time from injury to operation. A *P* value of >0.05 was regarded as not significant. All patient clinical and demographic data can be seen in Table 1.

### 2.2. Surgery Procedures

All patients in control and virtual group underwent X-ray and CT (LightSpeed Ultra 16; GE Medical Systems, Waukesha, WI, USA) examinations of the knee. The CT parameters were as follows: 120 kV, 300 mA, bone window, 1 mm layer thickness, and 0.625 mm layer interval. The fractures were evaluated from X-ray and CT before surgery.

In control group the patients were placed supine on the operating table with a lower limb tourniquet on the affected side. A lateral approach was preferred for Schatzker type III fractures. After adequate exposure of the fracture site, the menisci were conserved, the depressed fragments were elevated, and the articular surface was reduced anatomically. After maintaining the reduction temporarily by K-wires, the articular surface congruency was checked with the aid of fluoroscopy. Then the LISS plate was fixed on the lateral of the tibia.

In virtual group all scan data in DICOM format were imported to Mimics 10.01 software, and then image positioning, threshold-value segmentation, and dynamic segmentation were performed. Each image layer was subjected to selective editing and hole filling to remove redundant data and then subjected to smoothing. Next, 3D reconstruction was performed to create 3D models of the Schatzker type III tibial plateau fractures. The Mimics 10.01 software was used for the 3D measurement of tibial plateau fracture depression and to obtain key technical parameters for the surgery. Less invasive stabilization system (LISS) plates and screws were precisely measured with Vernier calipers in order to obtain data for designing the computer model. Some dates of the sample size were achieved from the LISS system plates (Synthes, Paoli, PA, USA). We performed 3D reconstruction of the LISS plates and screws according to the principle of point-line-face all-in-one, using the SolidWorks software. The 3D reconstructed model was exported in the  .stl format. Using the measurement results, we formulated an accurate and detailed preoperative plan, determined the degree of depression, position, and direction of the surgical windowing and bone mass of the graft, and simulated the reduction procedure. Fracture fixation with a steel plate was completed in Mimics 10.01 software. Then we performed actual windowing and poking reduction and internal fixation according to the parameters of virtual surgery.

The surgeries of both groups were performed by 2 same orthopaedic consultants.

### 2.3. Outcome Measures

We recorded the operation time, incision length, and blood loss in two groups. After the surgery we took X-ray examination of the internal fixation and fracture reduction. The independent Student's *t*-test was used to analyse the differences with operative time, incision length, and blood loss. A *P* value of <0.05 was regarded as significant. The virtual surgical time of all patients experienced the test of average ($\overset{-}{\chi }\pm S$). Statistical analysis was performed using the SPSS statistical package, version 15.0 for Windows.

## 3. Results

The 3D model of tibial plateau fractures reconstructed using Mimics 10.01 software was rapidly prepared, accurate, clear and intuitive as a guide for the formulation of preoperative plans. Internal fixation as reconstructed through SolidWorks was accurate and consistent with the actual fixation.

**Figure 1:**
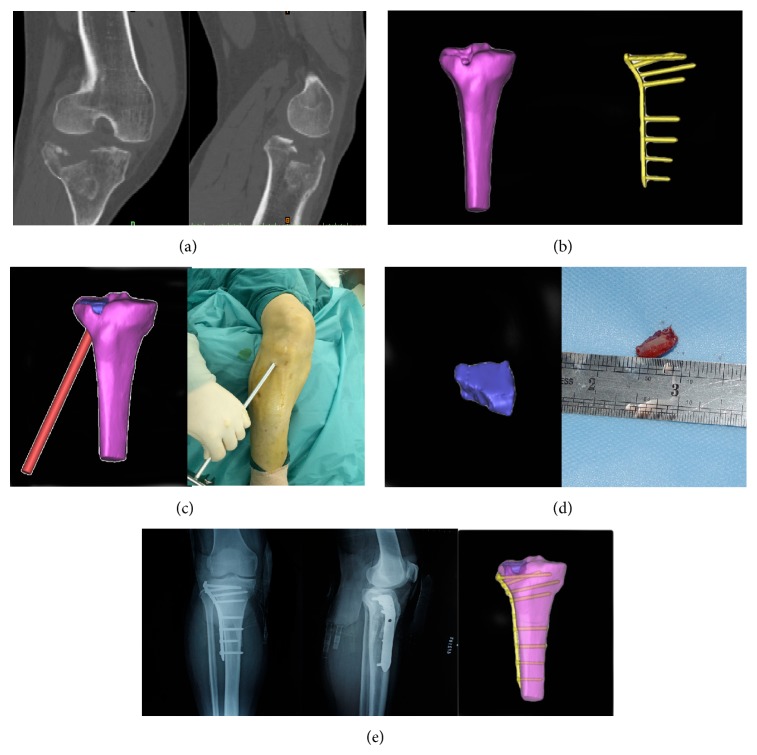
Comparison of virtual and actual surgeries. (a) Actual CT scan of a tibial plateau fracture. (b) Three-dimensional models of the same tibial plateau fracture and its internal fixation. (c) Creation of a surgical windowing and elevating reduction in the virtual and actual surgeries. Window position, window angle, and effective reduction were identical in the virtual and actual surgeries. (d) Virtual and actual bone grafts. (e) Postoperative radiographs were consistent with the virtual surgery and internal fixation.

In virtual group the virtual and actual surgeries were consistent in terms of fracture characteristics, position and angle of the surgical windowing, mass of the bone graft, selection of the steel plate, number and position of plate screws, and so forth. The postoperative radiographs were also consistent with the virtual surgery and internal fixation. A typical case was illustrated in Figure 1. The average time of the virtual operations was 43.3 ± 8.2 min (range, 32–60 min).

The mean operation time was 80.29 min (range, 74–90 min) in virtual group and 90.67 min (range, 85–100 min) in control group (*P* < 0.001). The mean incision length was 3.64 cm (range, 3–5 cm) and 6.44 cm (range, 5–8 cm) (*P* < 0.001). The mean blood loss was 95.71 mL (range, 80–120 mL) and 131.67 mL (range, 100–150 cm) (*P* < 0.001) (shown in Table 2).

## 4. Discussion

Tibial plateau fractures account for about 1% of all fractures and 8% of fractures in the elderly [5]. The principles for the treatment of tibial plateau fractures are as follows: anatomic reduction of the articular surface, restoration of the normal lines of force, reliable fixation, maximum protection of the soft tissue, and early functional exercise. With these treatment principles, most patients can eventually obtain good knee joint function. Successful surgical treatment of tibial plateau fractures requires the restoration of both articular surface integrity and the line of force of the lower limb [6–9]. Most authors agree that a depression or shift of >10 mm was an indication for the surgical elevation and profile recovery of the articular surface [10].

In Schatzker type III tibial plateau fractures, the most appropriate method to treat fracture depression was to create a surgical windowing and drill a hole in the relevant bone cortical area below the depressed joint. The method of surgical windowing and elevating reduction was used in the treatment of compression fractures in order to achieve anatomical reduction of the articular surface and avoid exacerbation of the damage due to direct prying or pulling. However, anatomical reduction of the articular surface may leave behind a large defect in the cancellous bone below the articular surface. This subchondral void can cause secondary fracture displacement and failure of fracture reduction. Therefore, subchondral bone grafting was required to successfully reduce the articular surface [11]. These fine surgical procedures necessitate accurate preoperative planning and evaluation. Three-dimensional reconstruction software can reveal the direction of the fracture line and fracture fragment displacement in a manner that can be intuitively understood by surgeons [12, 13]. In particular, it can clearly display crack fractures and limited depression fractures of the posterior aspect of the tibial plateau, thus overcoming an inadequacy of plain radiography. Three-dimensional reconstruction software can also rotate and slice the image and remove any bone tissue that occludes the target area to show the fracture from all angles. In addition to the position of the fracture and direction of fracture alignment, the reconstructed image can also show the size, shape, and direction of displacement and degree of depression for major fracture fragments and better visualize fractures of the posterior aspect of the tibial plateau than does plain radiography or CT [14, 15]. In this study, we constructed individualized preoperative 3D models of Schatzker type III tibial plateau fractures. Using these models, we conducted 2D and 3D measurements to determine surgical parameters such as the diameter of the tibial plateau, degree of fracture depression, position and angle of the surgical windowing, and mass of the bone graft. The personalized preoperative plans prepared using these parameters were accurate and detailed. The virtual surgery was consistent with the actual surgery.

With the development of the surgical “second-hit” theory in trauma control, medical workers are increasingly emphasizing minimal invasion. Using detailed preoperative plans, physicians are able to select suitable materials for internal fixation before the surgery, thereby avoid repeated comparisons and steel plate shaping during the surgery, and reduce the surgical time. In addition, the most appropriate surgical approach can be selected, and therefore, only a small incision is needed, which minimizes the damage to soft tissues as compared to conventional surgery, which requires a large incision. In this study preoperative virtual design and assisted operation showed significant advantages in operative time, incision length, and blood loss (*P* < 0.05) compared with direct surgery group. Thus, conventional orthopaedic surgery, which was heavily dependent on operator experience, can be replaced with a modern approach which was personalized, quantifiable, precise, and minimally invasive [16, 17]. Computer-aided surgical planning allows doctors to understand the surgical procedures required and determine the most appropriate method of internal fixation, thereby shortening the surgical time and reducing the rate of treatment failure.

However, virtual surgeries do have some limitations. The soft tissue around the fracture cannot be considered. Furthermore, the average time of virtual surgery, 43.3 min in this study, was too long. Consequently, further improvements in the “realness” of virtual surgeries and reduction of the time required for virtual surgery are required for the widespread clinical use of this technique.

## 5. Conclusions

Preoperative virtual surgery design was consistent with the actual surgery. It was superior to direct surgery in the aspect of operation time, length of cutting, and blood loss. But its long duration was a barrier to the clinical application of this technology.

## Figures and Tables

**Table 1:** Patient clinical and demographic data.

Parameter	Virtual group	Control group	*P* value
Number of patients	14	18	
Gender (male/female)	4/10	5/13	
Age in years (range)	46.1 (41–53)	46.8 (40–56)	0.45
Days from injury to operation (range)	4.5 (2–10)	4.3 (2–9)	0.94

**Table 2:** Comparisons of clinical result between two groups ($\bar{\chi }$ ± *S*).

Parameter	Virtual group	Control group	*P* value
Number of patients	14	18	<0.001
Operation time (min)	80.29 ± 4.81	90.67 + 3.03	<0.001
Incision length (cm)	3.64 ± 0.63	6.44 ± 0.98	<0.001
Blood loss (mL)	95.71 ± 13.99	131.67 ± 16.18	<0.001
